# Supplementary figures and images for: Oligomerization of the Clostridioides difficile transferase B component proceeds through a stepwise mechanism
Source: PLoS Pathog. 2025 Jul 21;21(7):e1013186. doi: 10.1371/journal.ppat.1013186 (PMC12303382; doi:10.1371/journal.ppat.1013186)

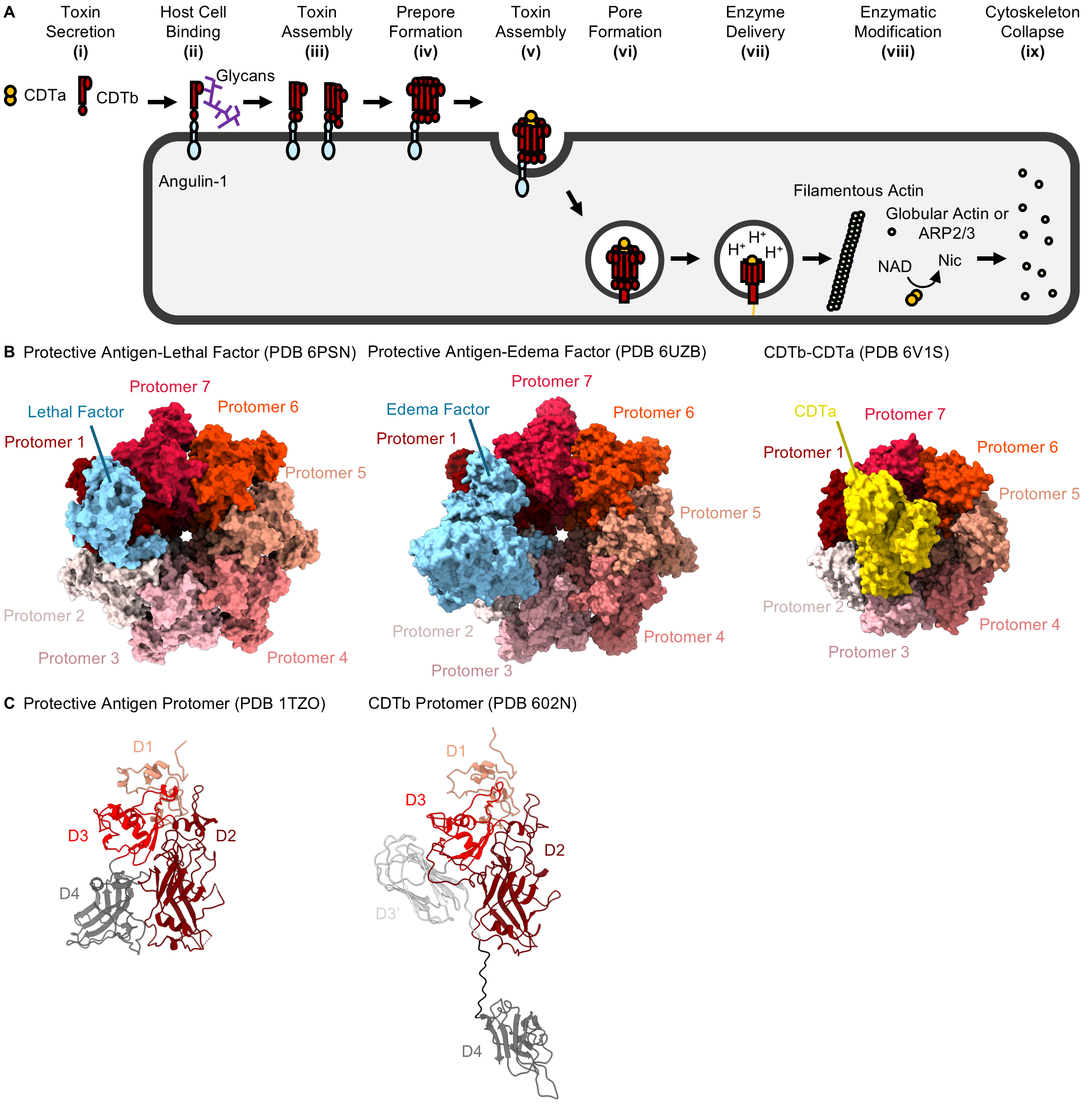

Supplement: S1 Fig — (A) The accepted model of CDT intoxication begins with toxin secretion at the site of infection (i). Once secreted, CDTb localizes to host cells through a receptor known as Angulin-1. CDTb has also been shown to interact with glycans though it is not clear what role this interaction plays during intoxication (ii). CDTb is then proteolyzed and oligomerizes (iii) to form a structure referred to as the ‘prepore’ (iv). The prepore binds a single copy of CDTa and enters cells via endocytosis (v). Within the endosome CDTb undergoes a structural rearrangement leading to the formation of a membrane-spanning channel or ‘pore’ (vi). In response to the environment of the maturing endosome, CDTa passes through the CDTb pore and into the host cell cytoplasm (vii). Inside the cell, CDTa modifies globular actin and the actin related protein 2/3 (Arp2/3, viii) leading to cytoskeletal collapse and a cell rounding phenotype (ix). (B) Structures of anthrax toxin consisting of lethal factor (left) and edema factor (center) in complex with protective antigen illustrate binding modes distinct from that of CDT (right). (C) Orientation of the receptor binding domain (D4) of anthrax toxin (left) compared to that of CDT (right). (TIF) [file ppat.1013186.s001.tif]

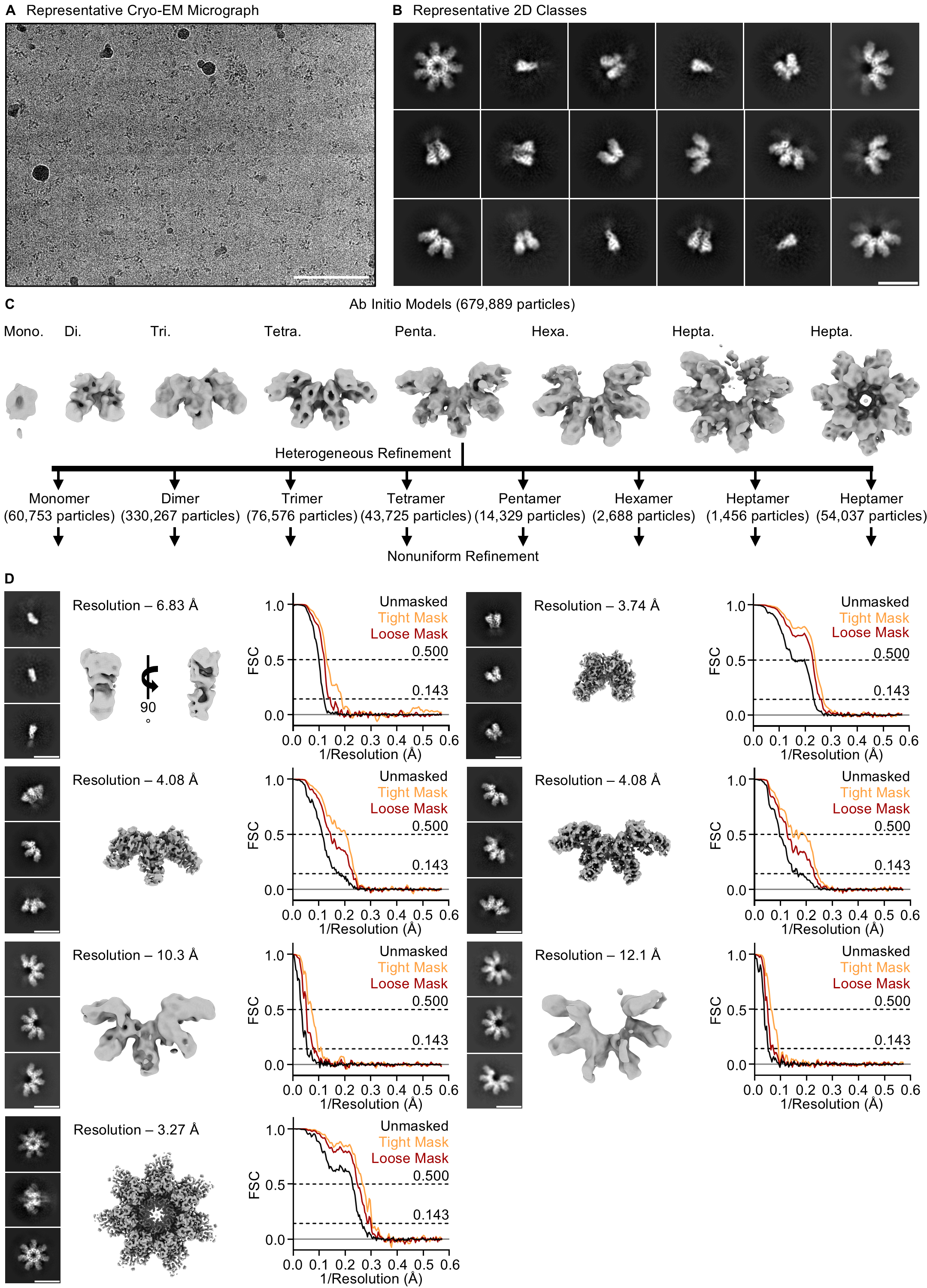

Supplement: S2 Fig — (A) A representative micrograph collected of the sample containing CDTb oligomeric intermediates. Scale bar is one µm. (B) Two-dimensional classification was used to sort particles into distinct oligomeric states. These particles were then used to generate eight ab initio models. Scale bar represents 100 Å. The scale is the same for all classes shown. (C) The ab initio models were refined to generate high resolution maps to be used as references for further refinement. All maps that were generated are shown below with representative two-dimensional classes illustrated on the left. Fourier shell correlation plots were used to estimate the global resolution of each map using a 0.143 cutoff as shown to the right of each map. (TIF) [file ppat.1013186.s002.tif]

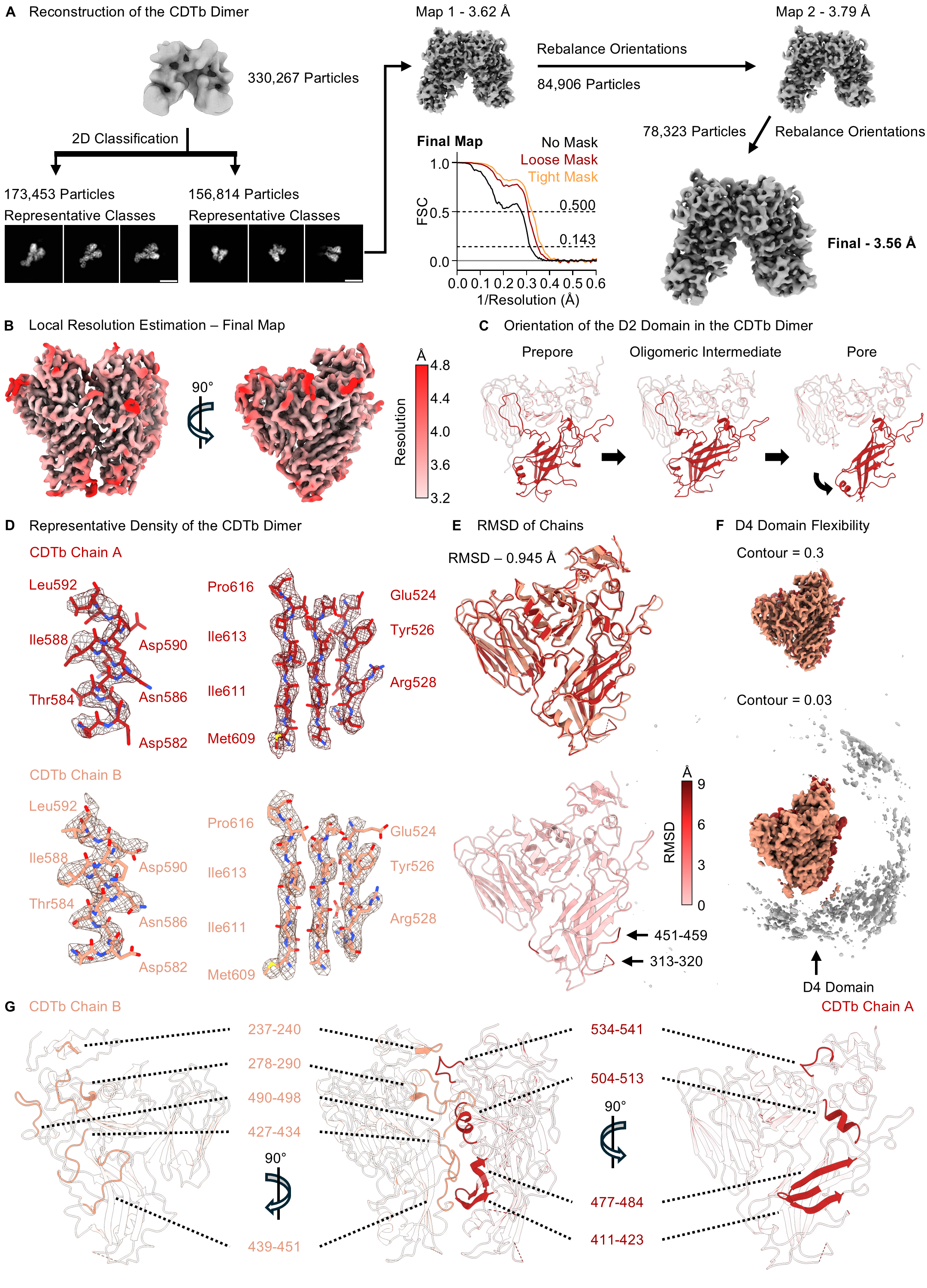

Supplement: S3 Fig — (A) Particles corresponding to the CDTb dimer were subjected to a final round of two-dimensional classification to separate structurally distinct particles. This dataset was then used to reconstruct a map of the CDTb dimeric assembly intermediate in three-dimensional space to a global resolution of 3.62 Å. Due to issues arising from preferred orientation, the resulting map suffered from anisotropic resolution and was subjected to iterative jobs to rebalance particle orientations within the dataset. The final map was reconstructed to a resolution of 3.56 Å as determined by Fourier shell correlation using a 0.143 cutoff. (B) The local resolution of the CDTb dimer is displayed on the final map. (C) The CDTb dimeric assembly intermediate is observed in a prepore-like configuration wherein the D2 domain adopts an outward facing conformation as opposed to the inward facing conformation reported in the structure of the CDTb pore. (D) Representative density of Chains A and B in the CDTb dimeric intermediate. (E) An overlay of Chains A and B is shown at the top with the backbone RMSD of all residues observed in both structures indicated at the bottom. (F) A low contour map of the CDTb dimer illustrating a lack of density corresponding to the CDTb D4 receptor binding domain (top). A high contour rendering of the same map indicates the presence of density that can be attributed to the D4 domain (bottom). (G) Residues involved in the interface that facilitates CDTb dimerization. Chain A is shown in scarlet and Chain B in salmon. (TIF) [file ppat.1013186.s003.tif]

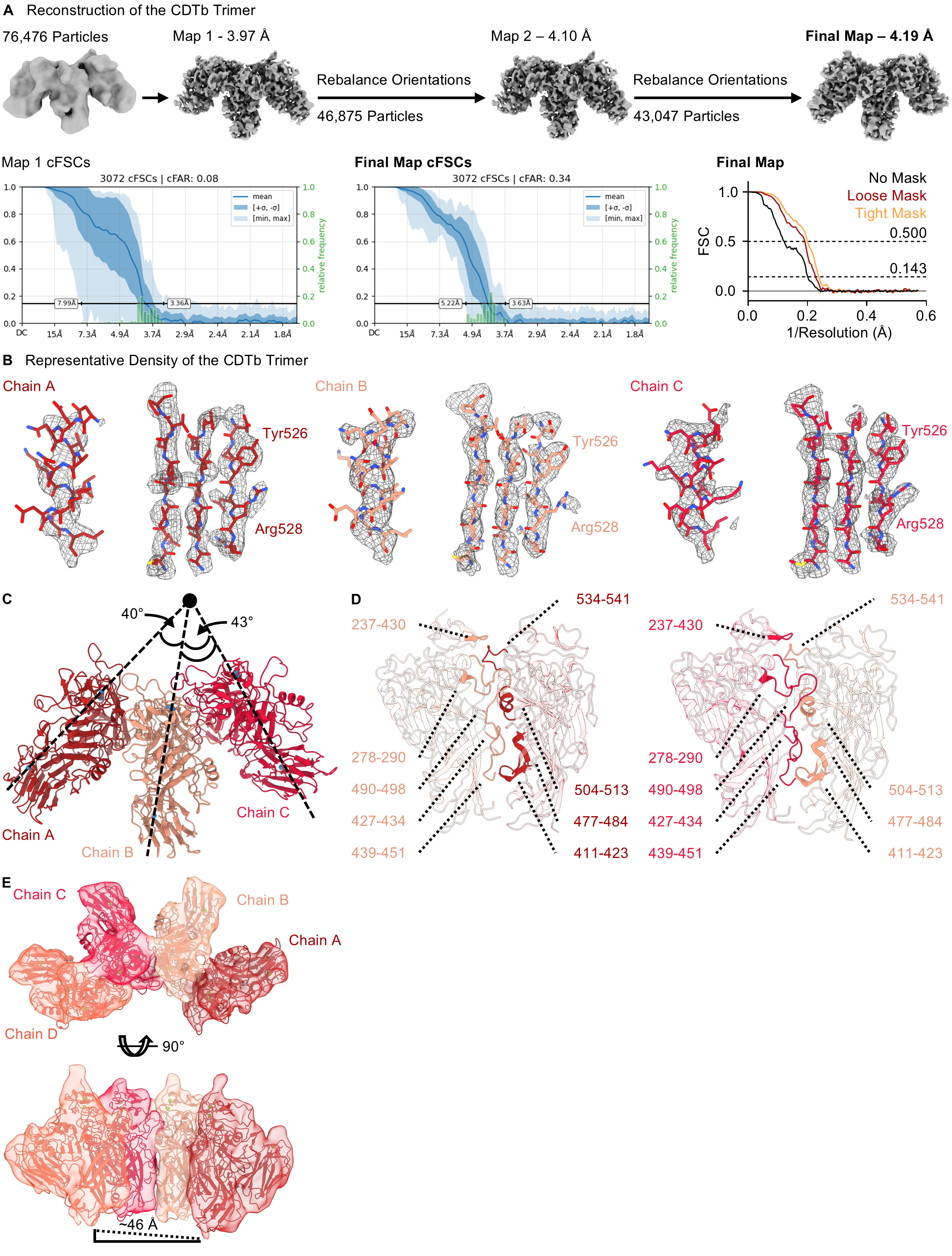

Supplement: S4 Fig — (A) An initial map of the CDTb trimeric assembly intermediate was reconstructed to 3.97 Å resolution. Particles were rebalanced iteratively to limit the effect of preferred orientation for this sample resulting in a final map of 4.19 Å resolution. (B) Representative density of the CDTb trimeric intermediate with the constructed model fit into the density. Chain A is depicted in scarlet, Chain B in salmon, and Chain C in red. (C) The relative orientations of Chains A and B and Chains B and C with respect to the central symmetry axis of the symmetric heptamer. (D) Residues facilitating interactions between Chains A and B (left) and Chains B and C (right). (E) The low-resolution map that was reconstructed of the CDTb tetrameric assembly intermediate. Chain A is shown in scarlet, Chain B in salmon, Chain C in red, and Chain D in peach. (TIF) [file ppat.1013186.s004.tif]

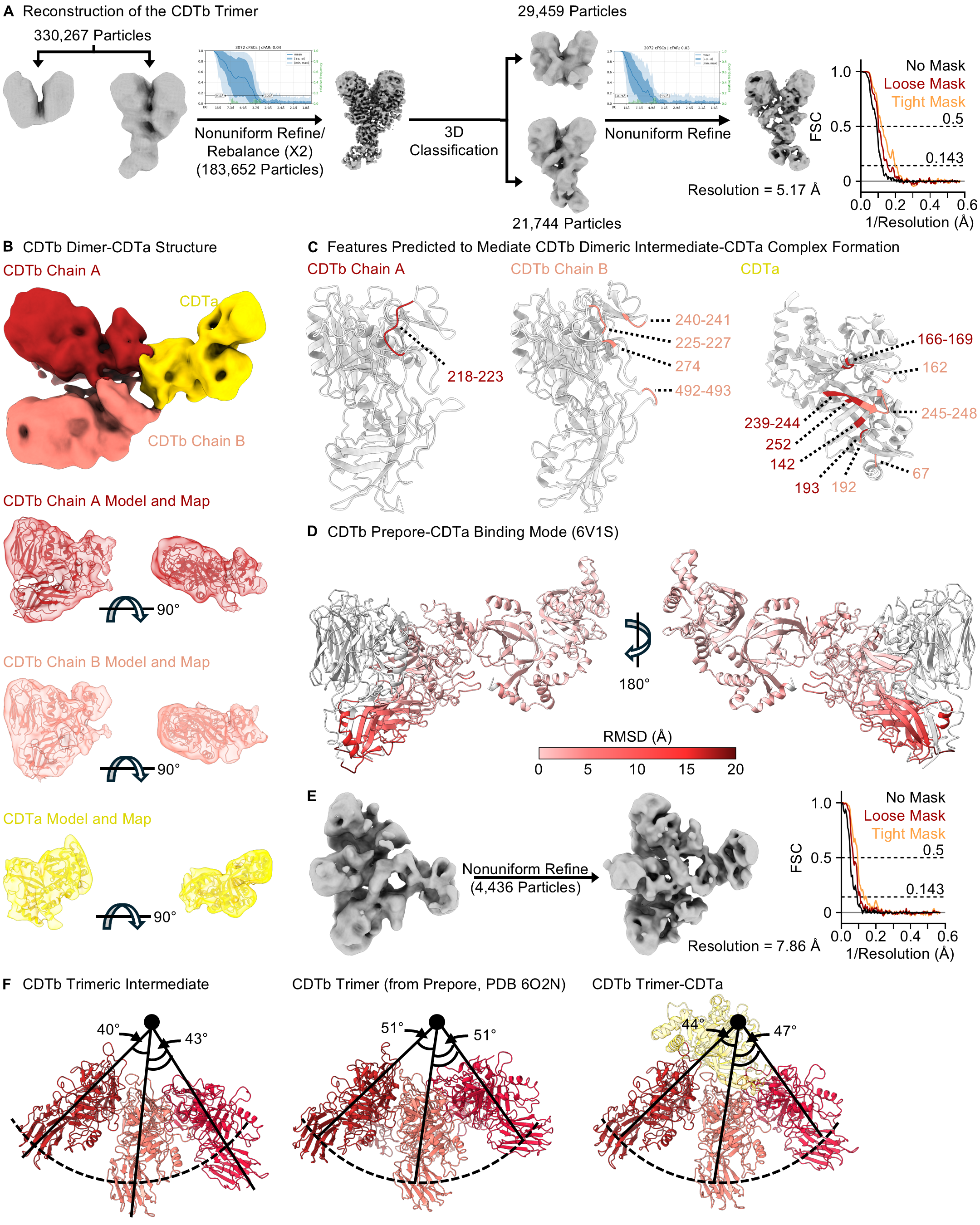

Supplement: S5 Fig — (A) Reconstruction of the CDTb dimeric intermediate bound to CDTa resulted in a map with a global resolution of 5.17 Å. Conical FSCs (inset) were used to define the resolution of the map in three dimensions and limit the influence of preferred particle orientation in the final map. (B) Density of the CDTb dimeric intermediate bound to CDTa colored to illustrate the location of CDTb Chain A (scarlet), CDTb Chain B (salmon), and CDTa (gold) is shown at the top. The fit of the generated model is shown for each chain below. (C) Residues predicted to be at the site of interaction between the CDTb dimer and CDTa. Residues interfacing with CDTb Chain A are shown in scarlet and residues interfacing with Chain B are shown in salmon. (D) A plot depicting the RMSD between the CDTb dimeric intermediate bound to CDTa and CDTa bound to the CDTb symmetric heptamer (PDB 6V1S). (E) Reconstruction of the CDTb trimeric intermediate in complex with CDTa led to the generation of a map resolved to 7.86 Å. (F) The relative orientations of Chains A (scarlet), B (salmon), and C (red) are illustrated for the apo CDTb trimeric intermediate (left), the trimer structure extracted from the CDTb symmetric heptamer (middle), and the CDTb trimeric assembly intermediate bound to CDTa (right). The relative orientations of Chains A, B, and C in the CDTb trimeric assembly intermediate more closely resemble that of the CDTb symmetric heptamer. (TIF) [file ppat.1013186.s005.tif]

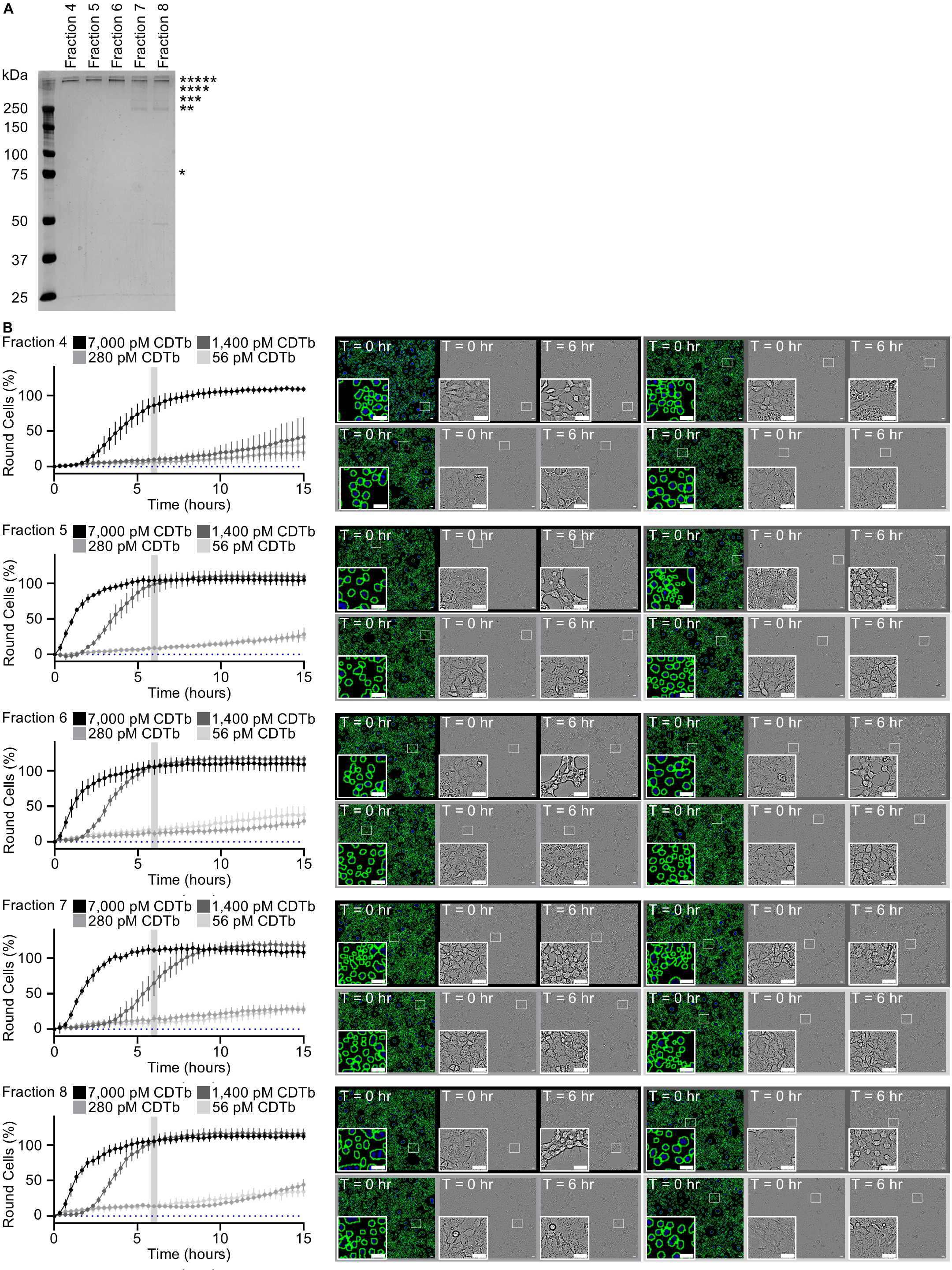

Supplement: S6 Fig — (A) CDTb assembly intermediates were isolated via size exclusion chromatography and analyzed by SDS-PAGE with silver staining. A * indicates the CDTb monomer while **, ***, ****, and ***** indicate unique molecular species with molecular weights corresponding to oligomeric assembly intermediates. (B) Enumeration of cellular intoxication assays for all five fractions are shown for the entire fifteen-hour assay (left). Error bars represent standard deviation. Representative images for each concentration assayed are shown on the right with the panels color-coded to reflect the concentration as depicted in the graph on the left. The first image in each series depicts the enumeration of nuclei with blue indicating Hoechst staining and green indicating the computational assignment of a nucleus. The remaining two images in each sequence are bright field images that illustrate the number of round cells at the onset of the experiment (middle) and after a six-hour incubation (right). (TIF) [file ppat.1013186.s006.tif]

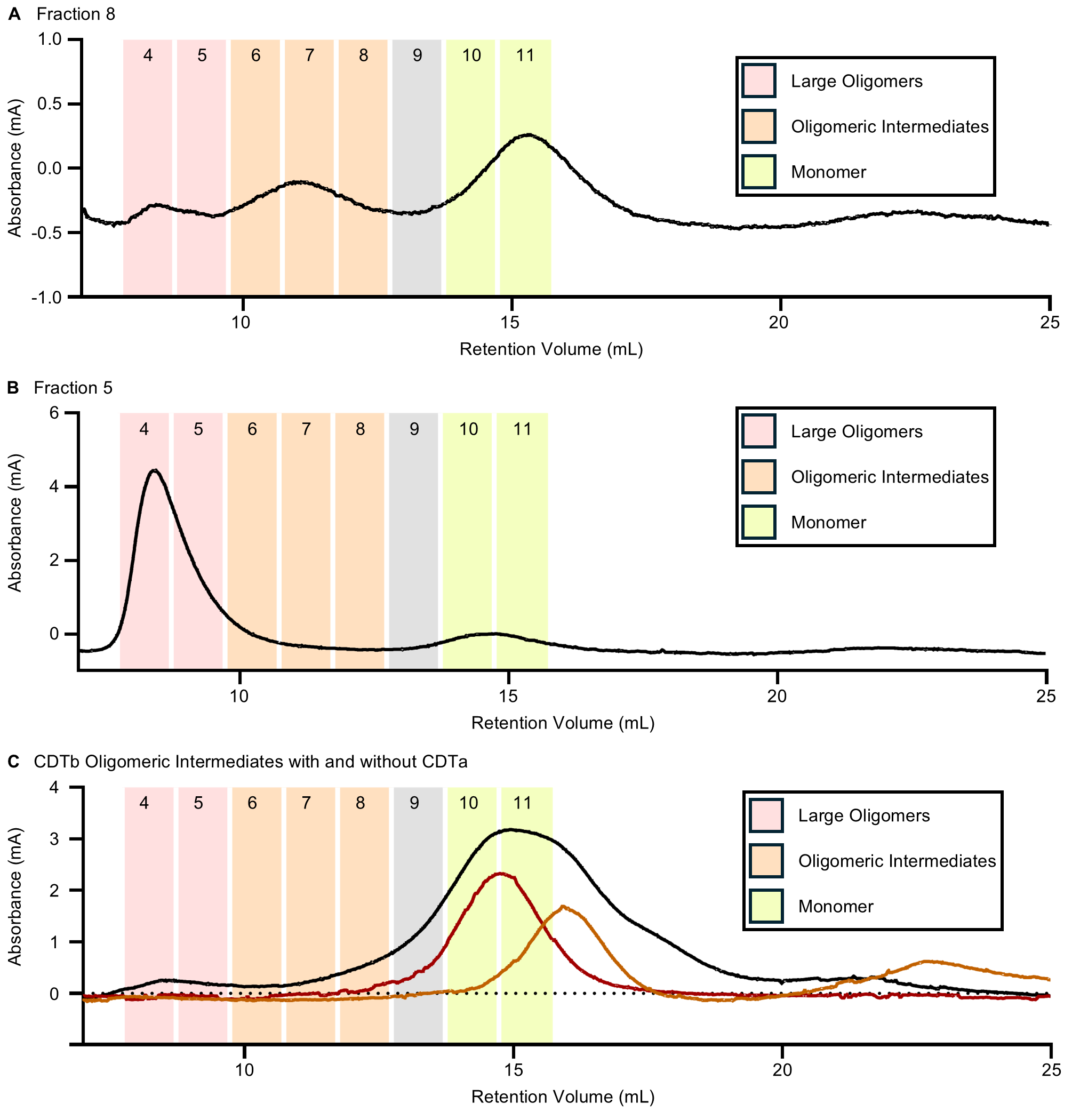

Supplement: S7 Fig — (A) The content of fraction eight was assessed via size exclusion chromatography and indicates the presence of large oligomeric particles (pink), oligomeric intermediates (orange), and the CDTb monomer (yellow). (B) The content of fraction five was assessed via size exclusion chromatography illustrating the presence of large oligomeric particles (pink) in this fraction. No oligomeric intermediates (orange) and a relatively low abundance of the CDTb monomer (yellow) were observed in this sample. (C) Size exclusion chromatography analysis of CDTa (orange), CDTb oligomeric intermediates (red), and CDTb oligomeric intermediates in the presence of CDTa (black). (TIF) [file ppat.1013186.s007.tif]

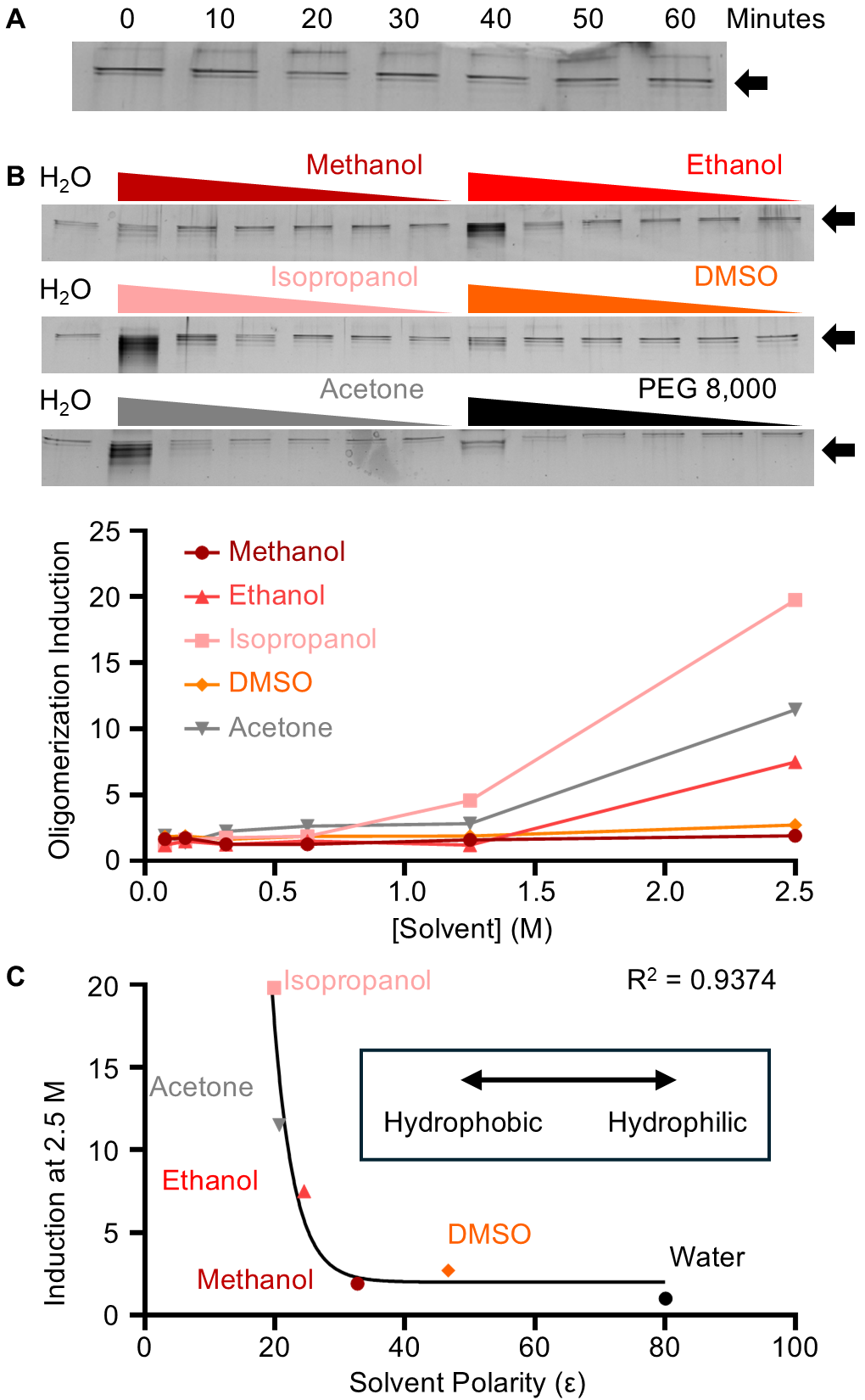

Supplement: S8 Fig — (A) In agreement with previous studies we note the oligomerization of CDTb is slow in vitro with little oligomer formation occurring after a 60-minute incubation. (B) Oligomerization was induced through the addition of various solvents and polyethylene glycol 8,000 (PEG 8,000). The concentration of the oligomer was quantified at various concentrations. DMSO – dimethyl sulfoxide. (C) The induction of oligomerization at 2.5 M solvent plotted against the polarity of the solvent and fit to an exponential decay function. DMSO – dimethyl sulfoxide. (TIF) [file ppat.1013186.s008.tif]

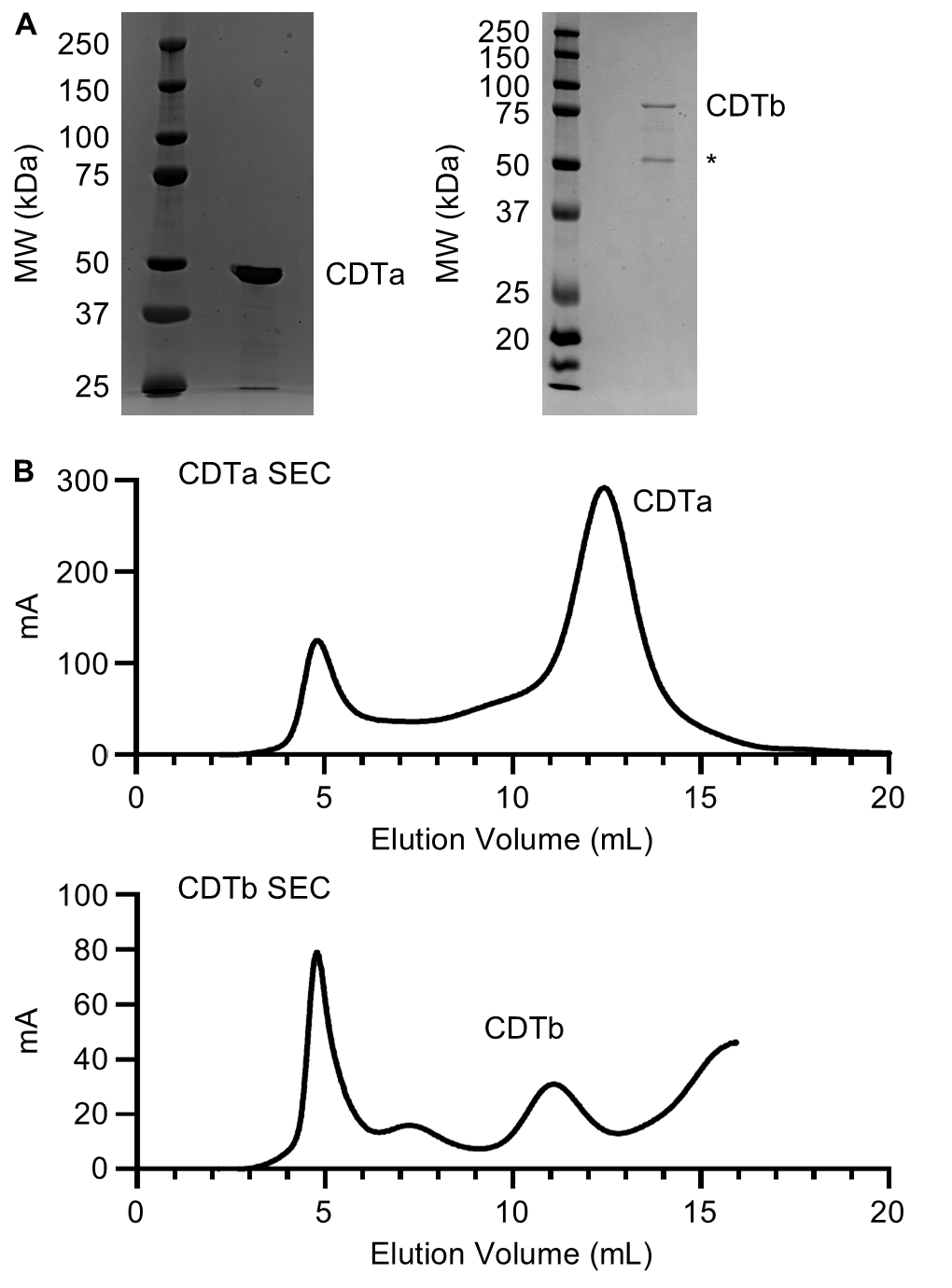

Supplement: S9 Fig — (A) SDS-PAGE analysis of representative samples of CDTa and CDTb stained with Coomassie blue. The * indicates a contaminating protein. (B) Size exclusion chromatography profiles of CDTa (left) and CDTb (right). (TIF) [file ppat.1013186.s009.tif]

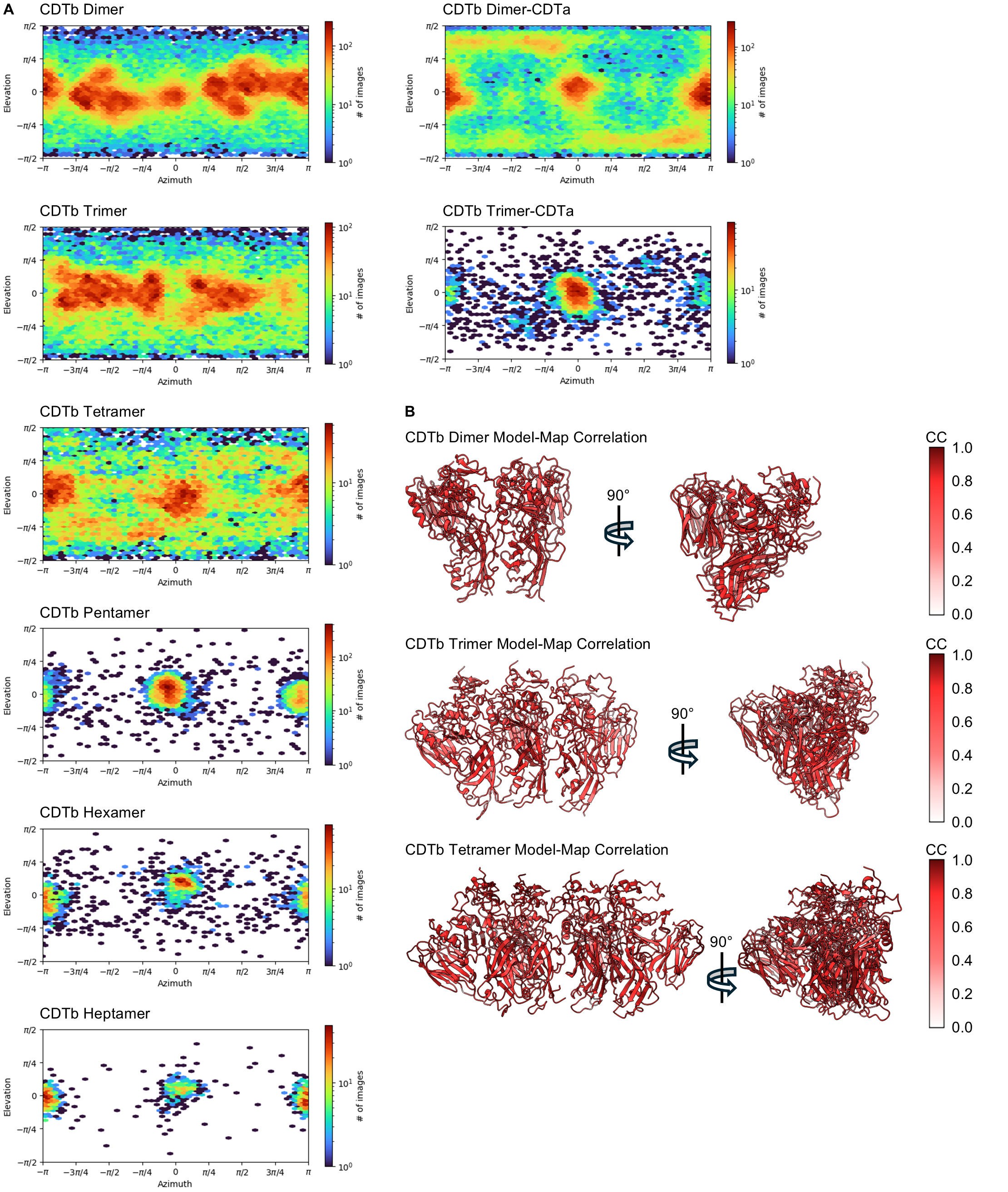

Supplement: S10 Fig — (A) The angular distribution profiles of all maps reconstructed during this study. (B) The per-residue correlation of the CDTb dimer, trimer, and tetramer oligomeric intermediates. (TIF) [file ppat.1013186.s010.tif]
