# Supplementary material for: Oligomerization of the Clostridioides difficile transferase B component proceeds through a stepwise mechanism
Source: PLoS Pathog. 2025 Jul 21;21(7):e1013186. doi: 10.1371/journal.ppat.1013186 (PMC12303382; doi:10.1371/journal.ppat.1013186)
Supplement: S1 Table — (PDF) [file ppat.1013186.s011.pdf]

## Supporting Table 1 – Cryo-EM Data Collection and Refinement Statistics

| **Data Collection and Processing** | **CDTb + CDTa** | | | | | | | | | |
|---|---|---|---|---|---|---|---|---|---|---|
| Voltage (kV) | 300 | | | | | | | | | |
| Electron dose ($e^-$/Å$^2$) | 50 | | | | | | | | | |
| Pixel size (Å) | 0.86 | | | | | | | | | |
| Defocus range | -0.5 to -1.5 | | | | | | | | | |
| Number of Movies | 6,344 | | | | | | | | | |
| Total Particle Images | 679,889 | | | | | | | | | |
| **Map Reconstruction** | **CDTb Monomer** | **CDTb Dimer** | | **CDTb Trimer** | | **CDTb Tetramer** | **CDTb Pentamer** | **CDTb Hexamer** | **CDTb Heptamer** | **CDTb Heptamer (symmetric)** |
| EMDB Identifier | EMD-48170 | EMD-48171 | EMD-48172 | EMD-48173 | EMD-48174 | EMD-48175 | EMD-48176 | EMD-48177 | | EMD-48178 |
| Initial Particle Images | 60,753 | 330,267 | | 76,576 | | 43,725 | 14,329 | 2,688 | 1,456 | 54,037 |
| Apo/CDTa Bound | Apo | Apo | CDTa Bound | Apo | CDTa Bound | Apo | Apo | Apo | Apo | Apo |
| Initial Particle Images | 60,753 | 156,814 | 173,453 | 72,140 | 4,436 | 43,725 | 14,329 | 2,688 | | 39,547 |
| Final Particle Images (no.) | 51,697 | 78,323 | 21,744 | 43,047 | 4,436 | 25,019 | 14,329 | 2,688 | | 39,547 |
| Map resolution (Å) | 6.83 | 3.56 | 5.17 | 4.19 | 7.86 | 7.01 | 10.32 | 12.05 | | 3.33 |
| Symmetry imposed | C1 | C1 | C1 | C1 | C1 | C1 | C1 | C1 | | C7 |
| FSC Threshold | 0.143 | 0.143 | 0.143 | 0.143 | 0.143 | 0.143 | 0.143 | 0.143 | | 0.143 |
| **Refinement** | | | | | | | | | | |
| PDB Identifier | | 9MDI | 9MDJ | 9MDL | 9MDN | 9MDP | | | | 9MDR |
| Model Resolution (Å) | | 3.5 | 5.2 | 4.1 | 8 | 6.8 | | | | 3.3 |
| FSC Threshold | | 0.143 | 0.143 | 0.143 | 0.143 | 0.143 | | | | 0.143 |
| Map Sharpening *B* Factor (Å$^2$) | | 165.8 | 204.5 | 196.5 | 356.9 | 611.4 | | | | 157 |
| Model Composition | | | | | | | | | | |
| Non-Hydrogen Atoms | | 8,139 | 11,358 | 12,237 | 15,456 | 16,316 | | | | 18,389 |
| Protein Residues | | 1,043 | 1,440 | 1,569 | 1,966 | 2,092 | | | | 2,352 |
| Ligands | | 6 | 6 | 9 | 9 | 12 | | | | 14 |
| *B* factors (Å$^2$) | | | | | | | | | | |
| Protein Residues | | 68.07 | 361.17 | 94.43 | 522.66 | 570.86 | | | | 75.07 |
| Ligands | | 70.81 | 254.82 | 100.11 | 326.40 | 493.13 | | | | 52.39 |
| **Validation** | | | | | | | | | | |
| MolProbity Score | | 1.72 | 1.84 | 1.81 | 1.92 | 2.15 | | | | 1.73 |
| Clashscore | | 4.80 | 9.04 | 5.22 | 8.51 | 12.22 | | | | 6.75 |
| EMRinger Score | | 2.33 | N/A* | 0.64 | N/A* | N/A* | | | | 2.31 |
| Rotamer Outliers (%) | | 0.76 | 0.00 | 0.00 | 0.00 | 0.00 | | | | 0.00 |
| Ramachandran Plot | | | | | | | | | | |
| Preferred (%) | | 92.4 | 94.9 | 90.8 | 92.7 | 90.0 | | | | 94.9 |
| Allowed (%) | | 7.6 | 5.10 | 9.2 | 7.3 | 10.0 | | | | 5.1 |
| Outliers (%) | | 0.0 | 0.0 | 0.0 | 0.0 | 0.0 | | | | 0.0 |
| Bond Deviation | | | | | | | | | | |
| Lengths (Å) | | 0.004 | 0.004 | 0.003 | 0.003 | 0.004 | | | | 0.003 |
| Angles (°) | | 0.813 | 0.812 | 0.771 | 0.784 | 0.885 | | | | 0.640 |

* EMRinger was not run for these datasets as it is not suitable for moderate to low resolution datasets.
